# First Report of Ventriculoperitoneal Shunt Infection due to *Cyberlindnera fabianii*

**DOI:** 10.1155/2015/630816

**Published:** 2015-11-04

**Authors:** Jonathan Baghdadi, Peera Hemarajata, Romney Humphries, Theodoros Kelesidis

**Affiliations:** ^1^Division of Infectious Diseases, Department of Medicine, David Geffen School of Medicine at UCLA, Los Angeles, CA 90095, USA; ^2^Department of Pathology & Laboratory Medicine, David Geffen School of Medicine at UCLA, Los Angeles, CA 90095, USA

## Abstract

Fungal infections in the central nervous system (CNS) are associated with significant morbidity and death. Transient fungemia in immunocompetent patients without any other risk factors for fungemia has been suggested as a possible mechanism that may lead to serious fungal ventriculoperitoneal (VP) shunt infections, but evidence is lacking. The clinical spectrum, diagnosis, and optimal therapy of *Cyberlindnera fabianii* infections remain to be determined. We describe the first case of CNS infection due to *C. fabianii* that occurred in an immunocompetent adult with a VP shunt. Spontaneous translocation with yeast that is not part of the normal gastrointestinal flora in the setting of ingestion of multiple servings of a fermentation product was the likely source from which *Cyberlindnera fabianii* gained entrance into the VP shunt system, causing meningitis in this patient. The authors conclude that, in view of the high morbidity associated with yeast infection of the CNS, long-term antifungal therapy should be strongly considered in cases where the VP shunt cannot be completely removed. Transient fungemia may lead to invasive disease in an immunocompetent host with VP shunt, even in the absence of any other risk factors for fungemia and even after remote placement of the VP shunt.

## 1. Introduction

Ventriculoperitoneal (VP) shunt is one of the most common neurosurgical procedures. Although infection is a common complication of shunt procedures, fungi are rarely implicated in VP shunt infections [1–4]. Transient fungemia and secondary colonization of VP shunts in the absence of other risk factors for fungemia have been suggested as a possible mechanism of fungal VP infections [5] but definite evidence is lacking. The heterothallic yeast* Cyberlindnera fabianii*, previously known as* Hansenula fabianii*,* Pichia fabianii*, and* Lindnera fabianii* [6], belongs to the phylum Ascomycota and is a member of the same order (Saccharomycetales) as other medically important yeasts such as* Candida* spp. Results from a recent phylogenetic analysis based on large and small subunit ribosomal ribonucleic acids (rRNA) and translation elongation factor-1*α* gene led to separation of the genus* Cyberlindnera* from its parental genus* Williopsis* [7]. Only 14 infections due to this organism have been reported [8–16]. Of the 14 cases, 10 involved fungemia [9–16] and 4 were limited to the genitourinary tract [8, 16]. Herein, we report the first case of central nervous system (CNS) infection due to* C. fabianii* that also suggests that transient fungemia in immunocompetent patients without any other risk factors for fungemia and with a remote history of VP placement may lead to serious fungal VP shunt infections.

## 2. Case Report

A 49-year-old Vietnamese-American woman with history of depression presented to our institution with confusion, headache, and difficulty in walking. Her medical history was significant for a ruptured anterior communicating artery aneurysm 10 years earlier that required craniotomy, clipping, and placement of a ventriculoperitoneal (VP) shunt. Four months prior to her presentation, she underwent a second craniotomy for clipping of a right middle cerebral artery aneurysm that was found on surveillance imaging. On presentation, she denied fever, chills, neck stiffness, photophobia, or head trauma. She was afebrile. The rest of her vital signs and physical examination, including neurologic examination, were unremarkable. Laboratory data was significant for a white blood cell count of 7810 cells/mm^3^ with 66.6% polymorphonuclear cells. Other laboratory data were within normal limits. Imaging (computed tomography) of the neck, chest, and abdomen showed hydrocephalus and the VP shunt in position without kinks.

Shortly after presentation, the patient underwent urgent neurosurgery for VP shunt revision. Close examination of the shunt during surgery revealed an obstructed valve, which was replaced. The intraventricular portion of the shunt could not be removed for inspection due to tissue scarring. Postoperatively, the patient became somnolent and was intubated for airway protection. Cerebrospinal fluid (CSF) studies from samples taken intraoperatively were significant for 29 red blood cells per mm^3^, 11 white blood cells per mm^3^ (33% neutrophils, 55% lymphocytes, and 12% monocytes), protein 65 mg/dL, and glucose 29 mg/dL. CSF Gram stain demonstrated budding yeast. Other studies, including serum and CSF cryptococcal antigen (ImmunoMycologics, Inc., Norman, OK),* Coccidioides* IgM and IgG antibodies (Meridian Bioscience, Inc., Cincinnati, OH), and bacterial and fungal blood cultures, were negative.

She was started empirically on intravenous liposomal amphotericin B at a dose of 5 mg/kg daily and oral flucytosine at a dose of 25 mg/kg four times daily on hospital day 1. Her mental status improved on antifungal therapy and she was extubated on hospital day 3. Though fungal CSF culture from hospital day 3 showed growth of a yeast after 10 days of incubation, CSF specimens collected on hospital days 5 and 7 were negative for microorganisms, both by fungal and Gram stain, and culture. A second neurosurgical procedure was performed, but the deep intraventricular portion of the VP shunt still could not be removed due to severe tissue scarring. Instead, that end was ligated, the remainder of the shunt was removed, and a new shunt was placed on the contralateral side.

**Figure 1:**
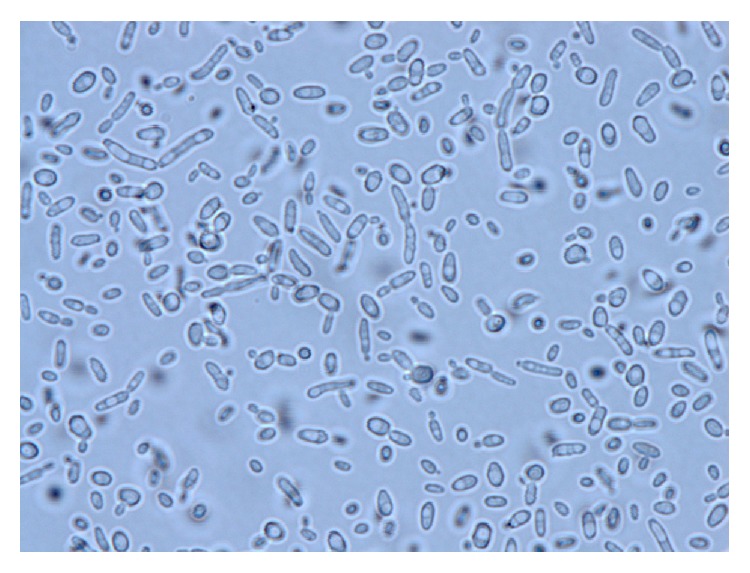
Wet mount preparation of* C. fabianii* from growth on IMA, demonstrating budding yeast cells and elongated pseudohyphae.

The yeast isolated from CSF collected during the first neurosurgical procedure demonstrated lush growth after 24 hours of incubation on inhibitory mold agar (IMA) [17] at 30°C. Two colony morphotypes (smooth and rough off-white colonies) were observed. Wet mount preparation from both morphotypes demonstrated budding yeast cells with pseudohyphae (Figure 1). Both morphotypes were identified by a Vitek MS Matrix-Assisted Laser Desorption/Ionization Time of Flight (MALDI TOF) mass spectrometer (bioMérieux, Inc., Durham, NC) as* Candida boidinii*. API 20C System (bioMérieux, Inc., Durham, NC) identification yielded profiles that were nondescriptive. A definitive identification of* C. fabianii* for both morphotypes was achieved through sequencing of the D1/D2 region of the large subunit of 28S ribosomal RNA gene and internal transcribed spacer (ITS) region [18]. Antifungal susceptibility testing was performed according to CLSI standards by broth microdilution in RPMI, in panels prepared in-house [19]. Antifungal minimum inhibitory concentrations (MIC) for fluconazole, flucytosine, and amphotericin B were 2.0, <0.12, and 2.0 mg/L, respectively.

Follow-up detailed history for possible environmental exposures revealed that the patient had ingested multiple servings of cơm rượu, a traditional Vietnamese dessert prepared by fermenting rice, 5–7 days before the onset of illness. The patient denied ingestion of raw milk and cheese.

The patient was discharged home after 11 days, still receiving intravenous liposomal amphotericin B and oral flucytosine. After 19 days of antifungals, she developed acute kidney injury and her regimen was changed to monotherapy with oral fluconazole. She developed painful cheilitis with fluconazole 800 mg daily and thus had her dose adjusted to 400 mg. At 6-month follow-up, she was feeling better and had returned to baseline function. She will continue fluconazole indefinitely to prevent recrudescence of her infection in the setting of retained VP shunt components.

## 3. Discussion

Fungal meningitis is an uncommon but serious condition affecting neurosurgical patients with VP shunts [1, 4]. Patients tend to present with fever and symptoms of shunt malfunction, such as headache, nausea, and altered mental status [1, 4]. In particular,* Candida* spp. have been identified as the cause of ~1% of VP shunt infections [1–4]. Up to 77% of* Candida* VP shunt infections develop within three months of shunt manipulation, suggesting inoculation of the organism during surgery [1–4]. Other risk factors include recent abdominal surgery, bowel perforation, use of broad-spectrum antibiotics, steroids, vascular catheters, prior or concurrent meningitis, and CSF leak [1, 4]. Transient fungemia in the absence of the above risk factors has been suggested as a mechanism that may lead to VP shunt fungal infection [5], but evidence is lacking. The current case demonstrates that transient fungemia with a yeast that is not part of the normal gastrointestinal flora may has occurred in the setting of ingestion of multiple servings of a fermentation product despite lack of known bowel disease. Thus, this case provides indirect evidence that supports the hypothesis that transient fungemia in immunocompetent patients without any other risk factors for fungemia may lead to serious fungal VP shunt infections, even with a remote history of VP placement.

*Candida fabianii* is an environmental yeast with similarities to* Candida* spp. Members of the genus* Cyberlindnera* exhibit phenotypic traits shared by members of the genera* Pichia*,* Issatchenkia*, and* Williopsis*, including production of ubiquinone CoQ-7 and inability to utilize methanol [7]. As with other heterothallic ascomycetous yeasts,* C. fabianii* is capable of sexual reproduction in which an ascus is formed containing small hat-shaped ascospores [20]. Despite being not typically performed in a clinical laboratory, organisms in this group can be induced to form ascospores using special sporulation media such as V8 agar and Gorodkowa agar [21]. Identification of this particular yeast species can be problematic [16]. Commercial identification platforms such as Vitek 2 with ID 32C system have been shown to incorrectly identify* C. fabianii* as* Candida pelliculosa* or* Candida utilis* [10]. Although MALDI-TOF has been shown in a recent report to be able to identify clinically relevant* Candida* spp., it failed to identify* C. fabianii*, whose spectral profile was not included in the Saramis reference database used with the Vitek MS [22]. Antifungal susceptibility testing may be performed on this organism using broth dilution method as for other yeasts, though there are currently no interpretive criteria [23].

Infections due to* C. fabianii* are extremely rare and have to date not involved the central nervous system. Details of the 14 reported cases are summarized in Table 1. Cases have been described in 9 neonates and 5 adults (average age of 48.6, range of 40–57). Three cases developed in individuals who were not known to be immunocompromised [9, 10, 14]. Three patients suffered from concurrent colitis [9, 11, 14]. Nine of ten (90%) fungemias developed in the setting of broad-spectrum antibiotics [9–16]. In all cases,* C. fabianii* was isolated by culture. In 13 of 14 cases (92.9%), species identification was confirmed by molecular sequencing. Antifungal susceptibility varies, with 10 of 14 (71.4%) cases involving isolates with MICs ≤ 2 *µ*g/mL for fluconazole [8–11]. Mlinarić-Missoni et al. recently described the susceptibility profiles of the 44 isolates (from six patients) of* C. fabianii* to amphotericin B, flucytosine, triazoles, and echinocandins and found a higher proportion of nonsusceptible isolates of* C. fabianii* for micafungin compared with the other tested antifungals [16]. No* in vitro* resistance to amphotericin B, flucytosine, fluconazole, itraconazole, or voriconazole was observed [16].

Treatments have been various, with amphotericin B, fluconazole, and caspofungin all used successfully. In two cases (14.3%), major surgery was required for source control [10, 13]. Microbiological and clinical cure was achieved in 11 cases (78.6%) [8, 10, 11, 13, 14, 16]. In the remaining three cases, the infected patient did not survive [9, 12, 15].

*Pichia* species are often found in raw milk, cheese, and traditional fermented products and a case of* Pichia anomala* (*Candida pelliculosa*) fungemia from ingestion of raw milk and yogurt has been published [24].* Candida fabianii *has been isolated from fermentation products [25] and home-made alcoholic beverages [26]. We suspect our patient acquired* C. fabianii *via ingestion of cơm rượu, a traditional Vietnamese dessert made from the fermentation of rice. We hypothesize that* C. fabianii *may have inoculated the peritoneal terminus of her VP shunt via translocation from the gut, either directly across the peritoneal space or by transient fungemia. This hypothesis is supported by the predilection of* C. fabianii *for neonates—whose gut mucosal immunity is undeveloped—and adults with colitis. A similar mechanism of infection has been proposed for* Saccharomyces cerevisiae*, which has caused fungemia in immunocompetent individuals ingesting the organism as a probiotic [27]. This case suggests for the first time that* C. fabianii *may lead to invasive disease in an immunocompetent host with a remote history of VP shunt placement after ingestion of commercially available fermentation products.

Given the rarity of* C. fabianii* infections, no guidelines are available to direct treatment. Decisions regarding antifungal therapy must be individualized. Amphotericin B and flucytosine were chosen initially, prior to determination of the species of the yeast and its antifungal susceptibility, based on clinical guidelines for central nervous system candidiasis [28]. By the time* C. fabianii* was identified, the patient had already recovered symptomatically, achieved microbiologic cure, and been transitioned to oral fluconazole for maintenance. Clinical studies of adults with cryptococcal meningitis have shown that a dose of fluconazole 400 mg daily achieves average CSF concentrations of 25.1 mg/L, a concentration at least 10 times higher than the MICs of our two isolates [29]. Further, there is a precedent for the use of fluconazole 400 mg daily for maintenance therapy in the management of cryptococcal meningitis [30]. In other instances of fungal meningitis, removal of indwelling devices such as VP shunts is considered standard of care [31, 32]. Unfortunately, components of our patient's infected VP shunt remain in place. Thus, she will likely require lifelong antifungal therapy. If recurrent infection develops, repeat susceptibility testing will need to be performed, as at least one case of* C. fabianii* has been reported to develop an elevated MIC to fluconazole and voriconazole during prolonged therapy [10]. In a case series of 6 cases of* C. fabianii* infection, fluconazole administration either prophylactically or therapeutically did not lead to resolution of the infection in 50% of the patients [16]. Thus, every effort should be made for complete removal of VP shunts infected with* C. fabianii* since in certain cases long-term oral suppression therapy with an azole may not be an option.

## 4. Conclusions

Clinicians should be aware of the clinical presentation, diagnostic methods, and therapeutic options for treatment of* C. fabianii* infection, as this organism may cause life-threatening infections including CNS infections. Transient fungemia may lead to invasive disease in an immunocompetent host with VP shunt, even in the absence of any other risk factors for fungemia and even after remote placement of the VP shunt. In view of the high morbidity associated with yeast infection of the CNS, long-term antifungal therapy should be strongly considered in cases where the VP shunt cannot be completely removed.

## Figures and Tables

**Table 1:** Cases of *C*. *fabianii* infection.

Reference/year	Age	Infection	Risk factor	Treatment	Clinical outcome
Dooley et al., 1990 [8]	57 y	*Hansenula fabianii* prostatitis	CLL, recurrent urethral self-manipulation	10-, 14-, and 28-d courses with ketoconazole, followed by AMB, duration NR.	Relapse off azole therapy. With AMB, the patient had lasting relief without recurrence of infection.

Valenza et al., 2006 [9]	46 y	*Candida fabianii* bloodstream, lung	Antibacterial therapy, enterocolitis	FLU for 15 d, followed by caspofungin daily for 7 d.	Disseminated intravascular coagulation, ischemic bowel, and persistent shock. Death at ICU, day 68.

Bhally et al., 2006 [11]	5 weeks	*Pichia fabianii* BSI	Prematurity, antibacterial therapy, and enterocolitis	AMB for 22 d. Vascular catheter removal on d 2.	Resolution of infection without sequelae at long-term follow-up.

Hamal et al., 2008 [10]	40 y	*Pichia fabianii* endocarditis	Congenital combined aortic incompetence of the mitral valve, recent craniectomy	FLU for 48 d, then VOR for 21 d, and then AMB for 35 d. Cardiac surgery after 28 d on AMB.	Recurrent stroke prior to cardiac surgery. Following surgery and AMB, no further evidence of infection.

Grenouillet et al., 2010 [12]	11 d	*Pichia fabianii* skin lesion, BSI, and empyema	Prematurity, antibacterial therapy, and premature rupture of membranes	FLU (duration NR) and then AMB and FC for 10 d (until death).	Gastrointestinal and tracheal hemorrhage. Oliguric renal failure. Death on 41 d of life.

Gabriel et al., 2012 [14]	53 y	*Lindnera fabianii* BSI	Antibacterial therapy, mesenteric ischemia	CAS for 19 d, followed by FLU for 91 d, and bowel resection.	Discharge from hospital on d 110.

Yun et al., 2013 [15]	47 y	*Lindnera fabianii* BSI	Antibacterial therapy, transplantation, steroids, and neutropenia	AMB (dose NR) for 8 d, followed by CAS for 14 d.	Multiorgan failure, shock, and death.

Wu et al., 2013 [13]	3 weeks	*Pichia fabianii* BSI	Low birth weight, broad spectrum antibiotics	FLU for 18 d.	Resolution of infection with discharge from hospital.

Mlinarić-Missoni et al., 2015 [16]	3.5 y/F	*C*. *fabianii* BSI	Leukemia, neutropenia, and antibacterial therapy	FLU for 5 d and then AMB for 14 d.	Clearance of fungemia after 3 d. Resolution of infection with discharge from hospital.

Mlinarić-Missoni et al., 2015 [16]	2 months/M	*C*. *fabianii* UTI	Hydronephrosis, surgery, and antibacterial therapy	FLU for 27 d, urinary catheter removal.	Clearance of culture after 11 d. Discharge from hospital.

Mlinarić-Missoni et al., 2015 [16]	Neonate/F	*C*. *fabianii* UTI	Gastroschisis, surgery, mechanical ventilation, parenteral nutrition, and antibacterial therapy	FLU for 27 d, urinary catheter removal, and CVC removal.	Clearance of culture after 5 d. Discharge from hospital.

Mlinarić-Missoni et al., 2015 [16]	Neonate/M	*C*. *fabianii* UTI	Hydronephrosis, surgery, parenteral nutrition, and antibacterial therapy	FLU for 30 d and then CAS for 10 d.	Clearance of culture after 10 d. Discharge from hospital.

Mlinarić-Missoni et al., 2015 [16]	Neonate/F	*C*. *fabianii* BSI	Intestinal atresia, surgery, parenteral nutrition, and antibacterial therapy	FLU for 15 days, CVC removal.	Clearance of culture after 7 d. Discharge from hospital.

Mlinarić-Missoni et al., 2015 [16]	Neonate/F	*C*. *fabianii* BSI	Pulmonary cyst, 740 g weight, antibacterial therapy, mechanical ventilation, and parenteral nutrition	FLU for 2 d and then CAS for 21 d.	Clearance of culture after 7 d. Discharge from hospital.

AMB: amphotericin B, BSI: bloodstream infection, CAS: caspofungin, CLL: chronic lymphocytic leukemia, CVC: central venous catheter, d: days, FC: flucytosine, FLU: fluconazole, ICU: Intensive Care Unit, ITRA: itraconazole, MIC: minimum inhibitory concentration, NR: not reported, VOR, voriconazole, and UTI: urinary tract infection.
